# Comparison of PAHs uptake by selected Monocotyledones and Dicotyledones from municipal and industrial sewage sludge

**DOI:** 10.1007/s11356-016-7130-2

**Published:** 2016-07-06

**Authors:** Barbara Gworek, Katarzyna Klimczak, Marta Kijeńska, Dariusz Gozdowski

**Affiliations:** 1 National Research Institute, Institute of Environmental Protection, Krucza 5/11 d, Warsaw, Poland; 2 Department of Soil Environment Sciences, Warsaw University of Life Sciences—SGGW, Nowoursynowska 159, Warsaw, Poland; 3 Department of Experimental Design and Bioinformatics, Warsaw University of Life Sciences—SGGW, Nowoursynowska 159, Warsaw, Poland

**Keywords:** PAHs, Root uptake, Sewage sludge, *Dicotyledones*, *Monocotyledones*

## Abstract

The study was focused on two goals: (i) the confirmation of the existence of a general relation between the content of polycyclic aromatic hydrocarbons (PAHs) in sewage sludge and in plants growing in it, regardless of the type and content of sewage sludge, and (ii) if so, the answer to the question whether the uptake of PAHs by plants depends on their type. To realize the set aims, the contents of PAHs in four differentiated plant species were measured, two belonging to the Monocotyledones and two belonging to Dicotyledones group, growing in municipal and industrial sewage sludge in two locations. All the investigations were carried out during the period of 3 years. The results clearly demonstrated that the uptake of PAHs by a plant depended on polyaromatic hydrocarbon concentration in the sewage sludge. The relation between accumulation coefficient of PAHs in plant material vs. the content of PAH in sewage sludge was of exponential character. The results indicate that in case of four- and five-ring PAHs, the root uptake mechanism from soil solution occurs, regardless of the type and origin of sewage sludge and the type of plant. For three-ring PAHs, we can assume for Monocotyledones that the root uptake mechanism occurs because we observe a significant correlation between the content of fluorene, phenanthrene, and anthracene in plant material and in the sewage sludge. For Dicotyledones, the correlation is insignificant, and in this case probably two mechanisms occur—the uptake by roots and by leaves.

## Introduction

Polycyclic aromatic hydrocarbons (PAHs) are environmental contaminants and constitute the largest class of suspected carcinogens and mutagens (van Metre and Mahler 2003). They appear in the environment and originate from natural sources (like forest fires), but most of their emission is connected with human activity (Benner and Gordon 1989; Garcia et al. 2012; Harvey et al. 2002; Lim et al. 1999; Weisman et al. 2010; Wild and Jones 1995) or, sometimes, human activity influences the mobility of PAHs in the environment (Zuijdgeest and Huettel 2012). They are known in particular as air contaminants occurring in places exposed to combustion smoke and situated near large urban centers (Arruti et al. 2012; De Nicola et al. 2005; Nielsen 1996). An additional source of emission of these compounds is cigarette smoking (Choi et al. 2010). They have special molecular structure—whole molecule is of aromatic character without substituents which is responsible for significant potential of interference with living organisms. The most studied and most known compound of the group is benzo[a]pyrene (Dutta et al. 2010), but it is to highlight that PAH compounds never exist alone—they always occur in a mixture, which was confirmed in many experiments (Mumtaz and George 1995).

It is proven that respiratory or dietary exposure to or skin contact with substances containing PAH mixtures causes lung cancer and tumors. That is why benzo[a]anthracene, benzo[a]pyrene, benzo[b]fluoranthene, benzo[j]fluoranthene, benzo[k]fluoranthene, dibenz[a,h]anthracene, and indeno[1,2,3-cd]pyrene were classified by the Department of Health and Human Services (DHHS) as known animal carcinogens (Mumtaz and George 1995). The International Agency for Research on Cancer (IARC) classified benzo[a]pyrene as human carcinogen (group 1), and some PAHs are classified as probable human carcinogens (group 2A) and or possible human carcinogens (group 2B) (IARC Monograph 2010).

As mentioned above, it is very difficult to assess the influence of PAHs’ presence in dietary exposure on living organisms. Nevertheless, the fact that they are carcinogenic for animals and due to some novel proofs that not only respiratory but also dietary exposure to PAHs may be carcinogenic for humans (Lee and Shim 2007; Yoon et al. 2007) raised the question whether PAHs accumulated in vegetables may develop different forms of cancer. The answer seems to be positive (Ashraf et al. 2013).

Considering the above, it is very important to define the pathway of the uptake of PAHs from sewage sludge to plants which is decisive for their content in plant material. Also of great importance is to find the relationship between polyaromatic hydrocarbon structure and the efficiency of their uptake by plants, i.e., which of the hydrocarbons are most easily accumulated by plants from a sewage sludge. Two mechanisms of the accumulation of PAHs by plants are taken under consideration. According to the first one, the transfer of PAHs from polluted atmosphere to plants occurs via particle-phase deposition on the waxy leaf cuticle or by the uptake from the gas phase through stomata (Duarte-Davidson and Jones 1996; Meudec et al. 2006). The second one assumes the root uptake from the soil solution and the liquid phase transfer in the transpiration streams (Duarte-Davidson and Jones 1996; Gao and Zhu 2004; Meudec et al. 2006; Yang and Zhu 2007; Zhu and Gao 2004).

There are numerous papers considering the influence of PAHs present in air on the development of cancer (Armstrong et al. 2004; Bosetti et al. 2007; Jung et al. 2010; Mastrangelo et al. 1996; Ravindra et al. 2001). Several regulations were prepared indicating the acceptable content of PAHs in the air, water, and soil. However, only very few studies take into account the influence of PAH content in plant and animal commodities on the human health. Also, a lack of norms regulating the content of PAHs in market products should be underlined.

Since the second half of the 1980s of the twentieth century (e.g., the Directive 86/278/EEC), there is a visible trend in the European Union jurisdiction to allow the utilization of sewage sludge, which always contains significant quantities of PAHs, as a natural fertilizer in the agriculture. This enhances the necessity to answer the question, if there is a correlation between the content of PAHs in sewage sludge and their concentration in a plant growing in it. The studies on the content of PAHs in sewage sludge and the changes of PAHs during sewage sludge composting were conducted by Oleszczuk (2007), but they are not devoted to the bioavailability of PAHs in sewage sludge. There are some studies about bioavailability of PAHs in soils (Guo et al. 2016; Lal et al. 2015), but it can be additional information only. The aim of our research was to check the influence of sewage sludge on PAH content in plants under natural conditions. Most studies on contaminants like PAHs are conducted under greenhouse conditions or in pots (Chen et al. 2016; Wieczorek et al. 2015). This study was conducted during 3 years on plants growing spontaneously on sewage sludge lagoon. Such approach can make the conclusions from the study more realistic and make the conclusions valuable for predicting possible contamination with PAHs in food or feed of plant origin.

The results of our previous research (Gworek et al. 2014) demonstrated the appearance of a strong correlation between the concentration of PAHs in sewage sludge and in plants growing in it.

The present study was focused on two goals: (i) the confirmation of the existence of a general relation between the content of PAHs in sewage sludge and in plants growing in it, irrespective of the type and content of sewage sludge, and (ii) if so, the answer to the question, whether the uptake of PAHs from the ground depends on the type of plant.

To realize the set aims, the contents of PAHs in four differentiated plant species were measured, two belonging to the Monocotyledones and two belonging to the Dicotyledones group, growing in municipal and industrial sewage sludge in two locations. All the analyses were carried out during the period of 3 years.

## Materials and methods

### Sampling

Two sewage sludge lagoons, one situated near a refinery-petrochemistry complex (location 1—L1) and one in an urban area (location 2—L2), were selected for this study. At each site, the samples of sewage sludge and of plant material growing in it had been collected for 3 years. In the sewage sludge and in plant material, the concentrations of 13 of the PAHs listed on US EPA specification (Zhou and Zhao 2014) were estimated.

### Characteristics of sampling sites and locations

#### Location 1—sewage-treatment plant

In the chosen location, there is one lagoon with sewage sludge of petrochemical origin from the integrated refinery-petrochemistry complex. The refinery part of the complex processes crude oil to fuels (gasoline, diesel oil, jet fuel, etc.), as well as to other oil fractions (e.g., boiler fuel) and to olefins (ethylene, propylene, C_4_ fraction), while the petrochemical sector produces a wide scale of basic chemicals (ethylene oxide, phenol, benzene, toluene, xylenes, etc.). During crude oil processing, various hydrocarbon fractions are formed, some of which as volatile organic compounds are contaminations emitted as the so-called “low emission” into the atmosphere. Others, such as stable organic contaminations, are collected on sludge lagoons and stored there.

In the refinery-petrochemistry complex, some technologies were adopted, allowing for the reduction of PAH content in the sewage sludge carried onto a lagoon. All wastewater is treated in a four-stage cycle: mechanical treatment; biological treatment and rainwater drainage; accelerators and rainwater drainage; and algal and reed ponds and rainwater drainage. Moreover, on the plant premises, there are 21 local pretreatment plants linked with respective units.

The sewage sludge originating from waste treatment in the refinery complex was stored on a field of a surface area amounting to ca. 10 ha. General characteristics of sewage sludge are as follows: pH 7.80–8.14, organic matter 29.08–35.20 %, and nitrogen content 2.44–2.51 %.

#### Location 2—municipal sewage-treatment plant

Location 2 is a small town of 11,000 inhabitants. The municipal sewage plant treated living household wastes and sewage from small-scale industry. All wastewater is treated in a two-stage process. The first phase is filtration, the second one is aeration tank. The sludge in the municipal sewage treatment plant in L2 was stored on four lagoons, each one of the surface area of 1500 m^2^. General characteristics of sewage sludge are as follows: pH 4.51–6.41, organic matter 39.21–75.20 %, and nitrogen content 0.15–0.88 %.

From each sampling site, the samples of sewage sludge and plants growing in it had been collected for 3 years. The sewage sludge from each lagoon was collected separately—from each lagoon, eight samples were taken and joined into a mixed sample.

The four plant species were found in both locations—couch grass (*Agropyron repens*), wood small-reed (*Calamagrostis epigejos*)*,* wild buckwheat (*Polygonum convolvulus*), and white goosefoot (*Chenopodium album*). The first two of them belong to the family of Monocotyledones and the last two to the Dicotyledones.

The plant samples included the dominating species of plants, as well as the accompanying species. According to the assumptions of the present study, the same plant species had to be collected in all study fields. Significant differentiation in the humidity of the base at various lagoons made it impossible in some cases.

### Methods

The samples used for the analyses were dried in a laminar chamber at room temperature (about 25 °C) during a few days for plants to 1 week for sludge. Consecutively, the dried substance was comminuted in a laboratory mill (plant samples) or grinded in a mortar (sludge samples). Sludge samples were subsequently screened on a sieve of 1 mm mesh. Due to the fact that the intention was to check if the usage of sewage sludge influenced PAH concentration in feeding staff, the concentration of PAHs was measured only in aerial parts of plants. Roots were not investigated. It allowed also to avoid the overestimation of PAH content caused by the absorption of PAHs onto roots.

#### The analyses of PAHs

The concentrations of 13 PAHs were evaluated, including the following:

three-ring (fluorene (Frn), phenanthrene (Ph), anthracene (A)); four-ring (fluoranthene (Ftn), pyrene (P), benzo[a]anthracene (BaA), chrysene (Ch)); five-ring (benzo[b]fluoranthene (BbF), benzo[k]fluoranthene (BkF), benzo[a]pyrene (BaP), dibenz[a,h]anthracene (DahA)); and six-ring (benzo[g,h,i]perylene (BghiP), indeno[1,2,3-c,d]pyrene (IP)).

The following procedure was applied for the analysis of sewage sludge and plant material samples: 10 g of air-dried and grinded material was mixed with 50 cm^3^ of methylene chloride and extracted in the presence of metallic copper in Soxtec apparatus during 3.5 h and subsequently washed during 1.5 h; the extracts were then concentrated to dried residue in a vacuum rotary evaporator; the dried residues were dissolved in 1-ml n-hexane portions and introduced to the column of 10 mm diameter filled with silica gel (10 cm layer) and basic alumina oxide (10 cm layer); the column was washed with methylene chloride-n-hexane solution of a concentration ratio gradually changing from 1:3 to 1:1, the eluate was concentrated to dry residue under nitrogen, and consecutively 2 ml of acetonitrile and 1 ml of n-hexane were added to the solid residue; and an aliquot of 10 μl sampled from the acetonitrile layer was diluted in 5 ml acetonitrile and 10 μl was injected by hand into the chromatograph.

The content of the examined polyaromatic hydrocarbons was determined by means of high-pressure liquid chromatography (HPLC) with photodiode (PDA) and fluorescent (FLD) detectors. Qualitative and quantitative analysis was performed using the FLD detector while the PDA detector was used mostly to confirm identification of PAHs in samples using UV spectra.

The qualitative analysis of each hydrocarbon using the FLD detector was based on retention time and different lengths of emission and extinction waves. Estimation of quantitative analysis was performed on a base of signals (peak area) by method of calibration curve (determination coefficient *r*^2^ was in the range 0.995–0.999) using capillary column SUPELCOSIL LC-PAH C18, S-5 μm, 15 cm × 4.6 mm. Ten microliters of sample was injected onto the capillary column, and separation was performed at 30 °C using gradient 50 % acetonitrile and 50 % water for 22 min to 100 %, followed by isocratic conditions for 36 min. The validation method was performed on reference materials of sewage sludge and the standard addition method for plants. Detection limit of the method was determined at 0.03 ng g^−1^ for all investigated PAHs analyzed together at the same time, which corresponds to 0.000180 μmol/kg for fluorene; 0.000168 μmol/kg for anthracene and phenanthrene; 0.000148 μmol/kg for fluoranthene and pyrene; 0.000131 μmol/kg for benzo[a]anthracene and chrysene; 0.000119 μmol/kg for benzo[b]fluoranthene, benzo[k]fluoranthene, and benzo[a]pyrene; 0.000108 μmol/kg for dibenz[a,h]anthracene; and 0.000109 μmol/kg for benzo[g,h,i]perylene and indeno[1,2,3-c,d]pyrene. The average relative standard deviation for chromatographic analysis was 10 %, and the average expanded uncertainty of method with 95 % confidence interval multiplied by the coverage factor k = 2 was about 30 %. The results were calculated taking into account the recovery value, which was in the range of 70–120 %.

The relation between the content of various PAHs in sewage sludge compared with the content in the plant growing in it was determined by the molar accumulation coefficient (m.a.c.) according to the following formula:

$$ \mathrm{m}.\mathrm{a}.\mathrm{c}.={\mathrm{n}}_{\mathrm{p}}/{\mathrm{n}}_{\mathrm{s}} $$

where n_p_ is the content of PAHs in plant material (μmol/kg) and n_s_ is the content of PAHs in sewage sludge (μmol/kg).

The corresponding concentration of PAHs, their uptake by plants, and the accumulation coefficient were expressed as molar magnitudes which allowed their direct use in further kinetic deliberations or biological activity description of an additive character.

#### Statistical analyses

For the evaluation of variability of PAH content, means and range for each species and sewage sludge were calculated. Relations between the accumulation coefficient of PAH in plant material and the content of PAH in sewage sludge were estimated with the use of power function model of regression (Y = aX^b^). The model was selected to maximize the coefficient of determination. The analyses were performed in Statgraphics 4.1 statistical software, and the figures were prepared in Microsoft Excel. Significance level for all analyses was set at 0.05.

## Results

In both chosen locations, the observations were carried out for 3 years and samples were collected from one field in location 1 and from four different fields in location 2.

The range of PAH concentration and their average values are presented in Table 1. The detected contents of PAHs for both locations under study differed remarkably. The sewage sludge in L1, located closely to the large industrial area, was cleaner and contained significantly lower amount of PAHs. A higher polyaromatic contamination was noted for L2, where only small industry appeared. This observation was most probably due to the much lower effectiveness of the municipal waste treatment station in L2 as compared with the industrial installation in L1. At the present state of the waste treatment procedures and technologies, the results of the above comparison denied the general belief that near-industrial areas are always more contaminated than municipal areas. The difference in the PAH content in both types of sewage sludge may be also connected to the significant difference in organic matter content in both types of sludge. The sewage sludge from L1 (industrial) had 29.08–35.20 % of organic matter and the sewage sludge from L2 (municipal) had 39.21–75.20 % of organic matter. There is a well-known relationship between the organic matter content in soils/sediments and probably sludge and the PAH contamination level.

**Table 1:** Means (μmol/kg) and range (min–max) for examined plant species and sewage sludge for both locations

PAH	Dicotyledones		Monocotyledones		Sewage sludge	
	*Chenopodium album* (μmol/kg)	*Polygonum convolvulus* (μmol/kg)	*Agropyron repens* (μmol/kg)	*Calamagrostis epigejos* (μmol/kg)	Location 1 (μmol/kg)	Location 2 (μmol/kg)
Frn	0.101	0.106	0.119	0.134	0.24	0.34
	(0.04–0.202)	(0.085–0.157)	(0.087–0.188)	(0.07–0.178)	(0.18–0.34)	(0.81–2.05)
Ph	0.201	0.233	0.328	0.276	0.79	1.43
	(0.103–0.318)	(0.194–0.32)	(0.241–0.393)	(0.163–0.328)	(0.53–1.17)	(0.81–2.05)
A	0.015	0.018	0.022	0.022	0.17	0.30
	(0.009–0.023)	(0.009–0.036)	(0.014–0.042)	(0.007–0.036)	(0.01–0.43)	(0.11–0.60)
Ftn	0.127	0.125	0.177	0.142	0.71	3.14
	(0.067–0.174)	(0.094–0.171)	(0.118–0.23)	(0.097–0.176)	(0.30–1.51)	(0.71–4.71)
P	0.075	0.116	0.088	0.073	0.59	2.1
	(0.048–0.112)	(0.051–0.232)	(0.065–0.142)	(0.043–0.095)	(0.12–1.26)	(0.63–3.65)
BaA	0.008	0.011	0.017	0.007	0.15	1.2
	(0.007–0.012)	(0.005–0.027)	(0.008–0.038)	(0.003–0.01)	(0.04–0.36)	(0.27–3.01)
Ch	0.020	0.015	0.025	0.017	0.15	1.59
	(0.014–0.028)	(0.009–0.024)	(0.01–0.053)	(0.009–0.024)	(0.06–0.33)	(0.41–3.80)
BbF	0.013	0.010	0.013	0.011	0.64	2.1
	(0.009–0.019)	(0.007–0.013)	(0.008–0.018)	(0.005–0.017)	(0.50–0.74)	(0.36–3.80)
BkF	0.005	0.004	0.006	0.005	0.22	0.91
	(0.004–0.007)	(0.002–0.005)	(0.004–0.009)	(0.002–0.008)	(0.18–0.28)	(0.15–1.94)
BaP	0.010	0.007	0.014	0.008	0.54	1.83
	(0.008–0.014)	(0.004–0.009)	(0.006–0.027)	(0.002–0.014)	(0.41–0.66)	(0.32–3.26)
DahA	0.002	0.002	0.003	0.003	0.25	0.34
	(0.001–0.003)	(0.001–0.005)	(0.001–0.007)	(0.001–0.004)	(0.06–0.45)	(0.03–0.93)
BghiP	0.007	0.004	0.009	0.006	1.45	1.49
	(0.003–0.01)	(0.003–0.005)	(0.004–0.014)	(0.003–0.009)	(0.34–2.01)	(0.27–2.84)
IP	0.005	0.005	0.006	0.005	0.73	1.43
	(0.003–0.007)	(0.003–0.007)	(0.005–0.008)	(0–0.008)	(0.31–1.04)	(0.23–2.58)

The determination of the content of PAHs in plants growing in the two investigated sewage sludge lagoons was the next step of our investigations. The four plant species were found in both locations—couch grass (*Agropyron repens*), wood small-reed (*Calamagrostis epigejos*)*,* wild buckwheat (*Polygonum convolvulus*), and white goosefoot (*Chenopodium album*). The first two of them belong to the family of Monocotyledones and the last two to the Dicotyledones.

The range of PAH concentration in plant material for each particular species and their average values are demonstrated in Table 1. Phenanthrene was present in dominant amounts in all analyzed plant materials, irrespective of the content of PAHs in sewage sludge. The content of phenanthrene in plants fluctuated from 0.185 μmol/kg (for *Chenopodium album* collected in L2) to 0.348 μmol/kg (in *Agropyron repens*, collected in L2). Furthermore, the content of phenanthrene in the plant material seemed not to be related to the content of phenanthrene in the sewage sludge. The amount of phenanthrene in sewage sludge from L2 was two times higher than the one in L1 (1.44 μmol/kg in L2 in comparison to 0.79 μmol/kg of phenanthrene in sewage sludge in L1), while the content of it in *Agropyron repens* and *Calamagrostis epigejos* was higher for the plant material collected in L2 and, oppositely, the content of phenanthrene in *Chenopodium album* and *Polygonum convolvulus* was higher for plant samples collected in L1.

In most cases, the second most widespread PAH in plants was fluoranthene (0.115 μmol/kg in *Chenopodium album* collected in L2 to 0.199 μmol/kg in *Agropyron repens*, collected in L2). The content of dibenz[ah]anthracene in plant material was the lowest in the entire series of PAHs (from 0.001 μmol/kg in *Calamagrostis epigejos* collected in L1 to 0.003 μmol/kg in *Polygonum convolvulus* collected in L1).

**Fig. 1:**
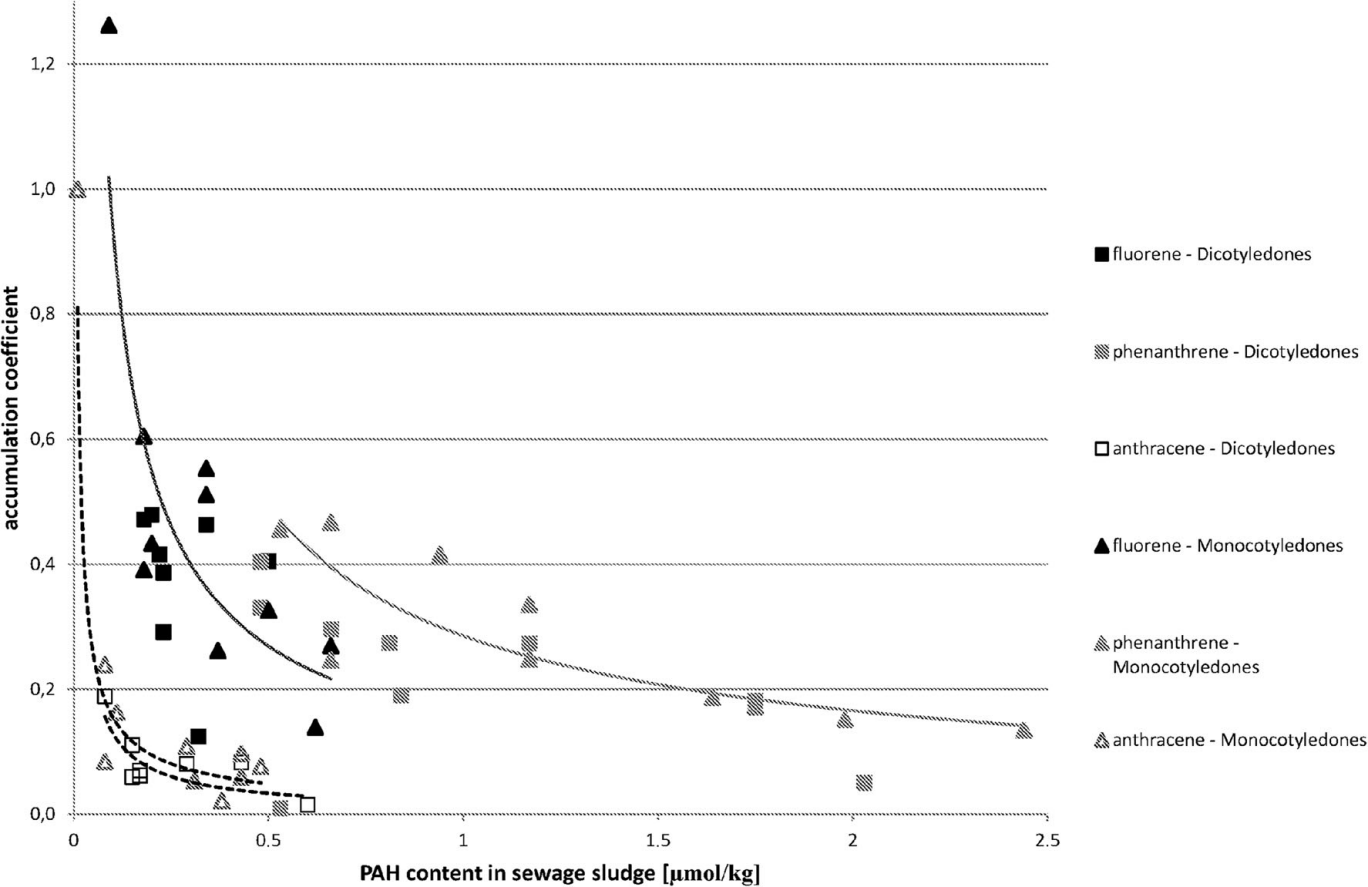
The correlations between accumulation coefficient of PAH in plant material vs. the content of PAH in sewage sludge for three-ring hydrocarbons (μmol/kg)

**Fig. 2:**
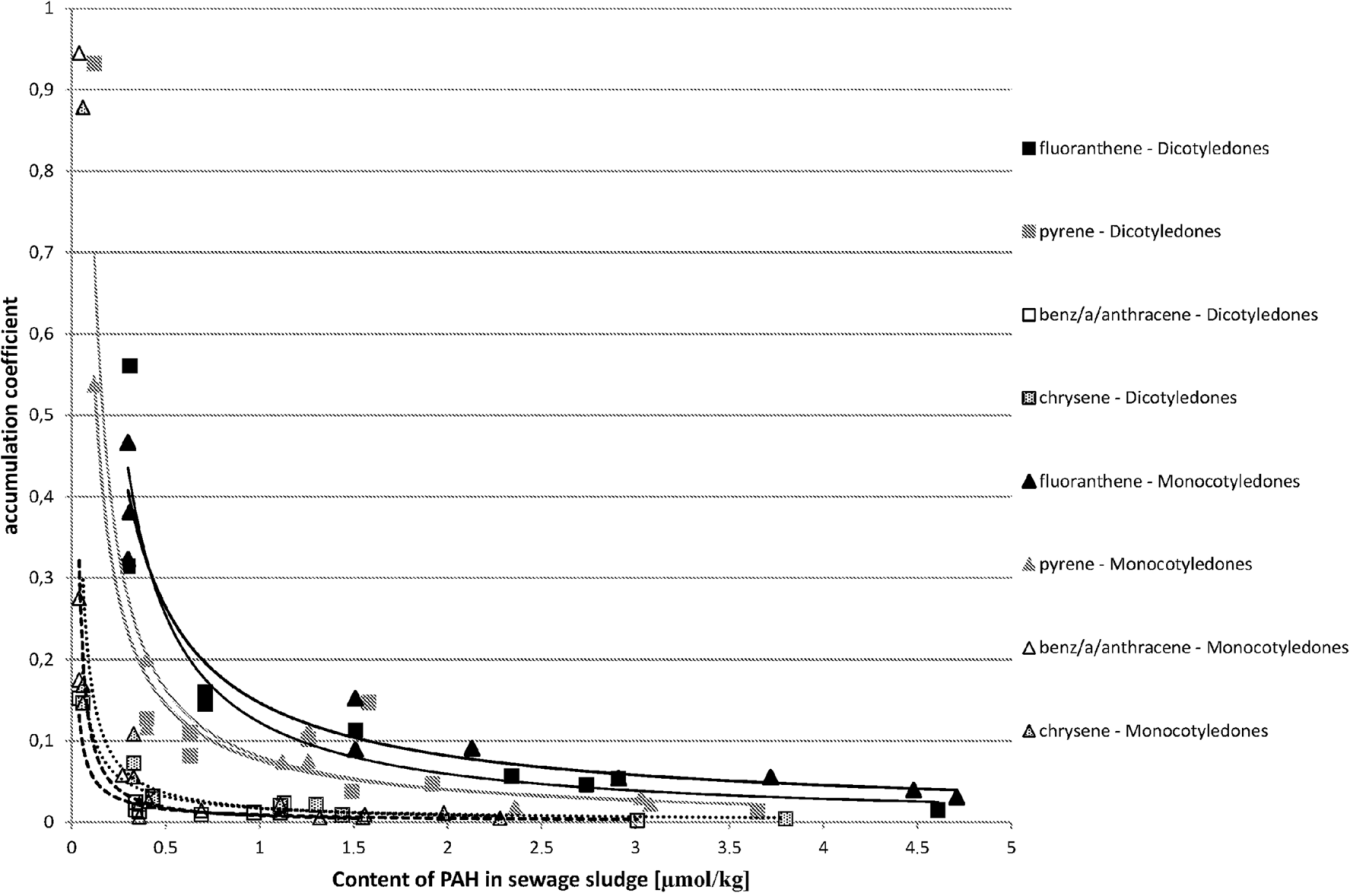
The correlations between accumulation coefficient of PAH in plant material vs. the content of PAH in sewage sludge for four-ring hydrocarbons (μmol/kg)

**Fig. 3:**
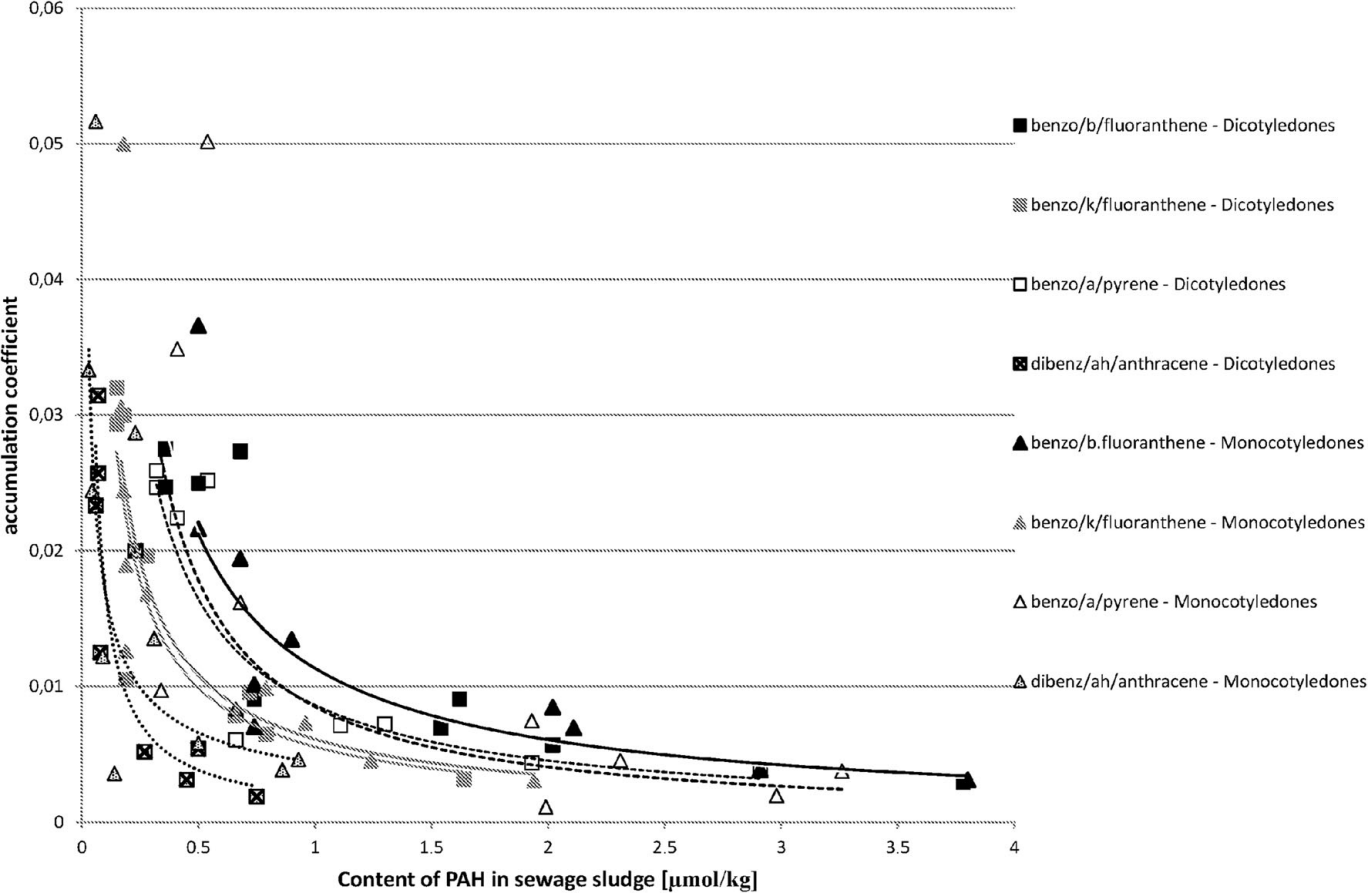
The correlations between accumulation coefficient of PAH in plant material vs. the content of PAH in sewage sludge for five-ring hydrocarbons (μmol/kg)

**Fig. 4:**
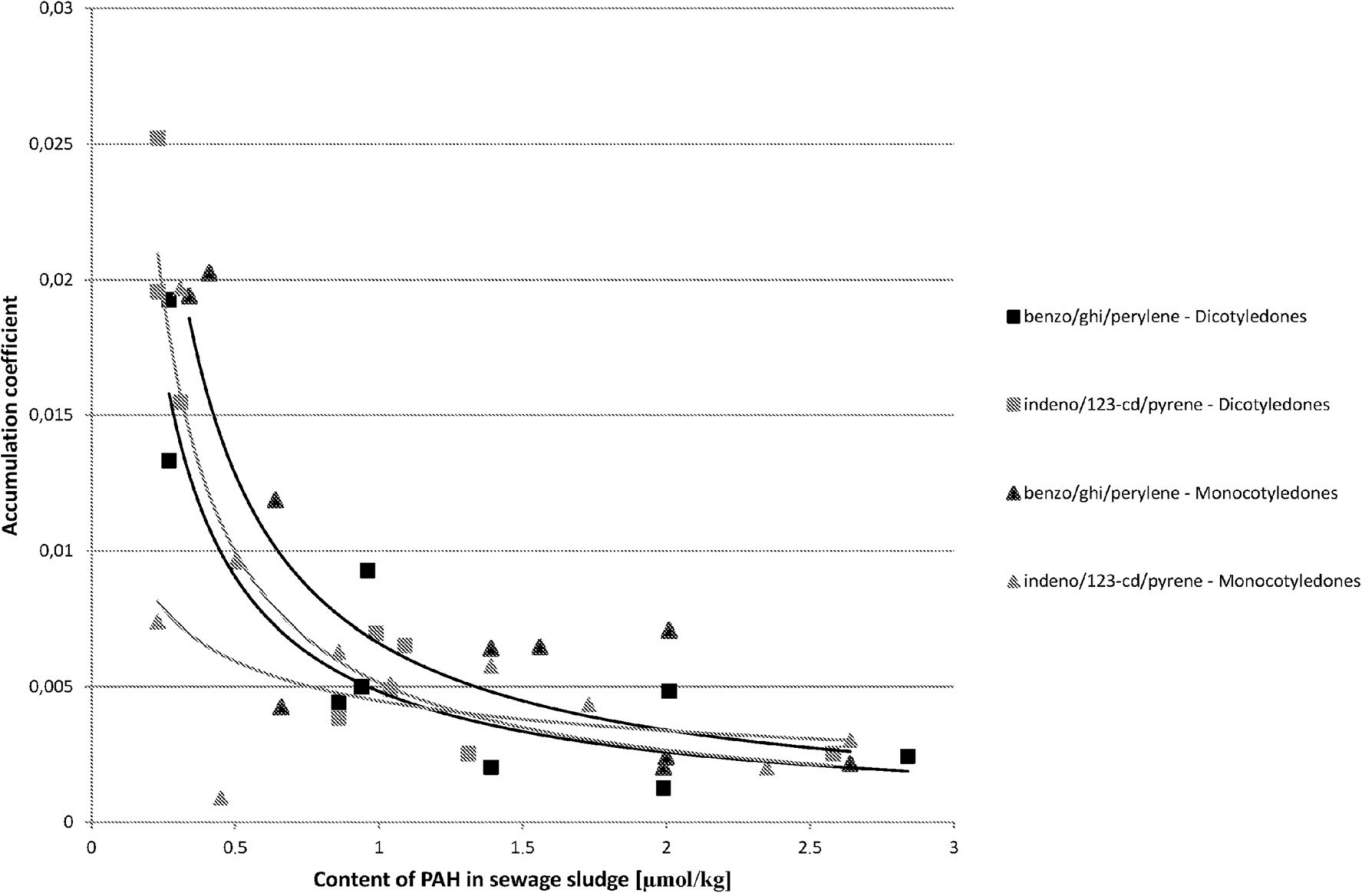
The correlations between accumulation coefficient of PAH in plant material vs. the content of PAH in sewage sludge for six-ring hydrocarbons (μmol/kg)

The analysis proved that in most cases there is a correlation between the content of PAHs in sewage sludge and in the plant material growing in it. The relation between the coefficient of PAH accumulation in plant material vs. the PAH content in sewage sludge is demonstrated in Fig. 1 for three-ring hydrocarbons, in Fig. 2 for four-ring hydrocarbons, in Fig. 3 for five-ring hydrocarbons, and in Fig. 4 for six-ring hydrocarbons. For all groups of hydrocarbons, the relation between PAH accumulation coefficient in plant material vs. the PAH content in sewage sludge was of exponential character.

## Discussion

One of the questions formulated at the beginning of the experiment was if it is possible to predict and assess the estimated content of PAHs in plants knowing the content of PAHs in sewage sludge and if it is possible to compare the results irrespective of the type of sewage sludge, its origin, and its characteristics. That is why the results are presented together, irrespective of the municipal or petrochemical origin of sewage sludge. Both types of sewage sludge had different characteristics, which could affect the bioavailability of PAHs. There were several studies conducted in different types of soil (Lal et al. 2015) suggesting the influence of soil characteristics on PAH bioavailability. The studies conducted by Guo et al. on soils did not gave a clear answer (Guo et al. 2016). There is lack of studies conducted on sewage sludge. In our research, we did not observe the effect of sewage sludge properties on accumulation coefficient. The results of the regression analysis between PAH accumulation coefficient in plant material vs. the PAH content in sewage sludge are shown in Table 2. *P* values below 0.05 indicate the existence of a significant correlation—for Monocotyledones, it appears for each PAH except indeno[123-cd]pyrene. For seven PAHs, the *P* value was below 0.001. In case of Dicotyledones, the *P* values below 0.05 have been established for all four-, five-, and six-ring hydrocarbons. In the three-ring PAH series, for anthracene the estimated *P* value was 0.030, which means that there is a significant correlation, but for fluorene and phenanthrene the *P* values were remarkably higher, 0.569 and 0.781 accordingly. As it was remarked earlier, two possible uptake mechanisms or their coexistence could be taken under consideration (Duarte-Davidson and Jones 1996; Gao and Zhu 2004; Meudec et al. 2006; Yang and Zhu 2007; Zhu and Gao 2004). It is very difficult to establish the primary source of PAH uptake by plants (Wieczorek et al. 2015). Even for plants growing in polluted soil, the mechanism of adsorption from gas-phase air may be a main source of PAHs, especially for three-ring volatile compounds (Kaćalkova and Tlustoš 2011). Also, Kipopolou et al. reported the increase of lighter PAHs in above-ground parts of plants in summer (Kipopoulou et al. 1999). It was also demonstrated by Oishi that apart from the mechanism, where the plant takes up gaseous PAHs via its stomata, also the absorption of dissolved PAHs by leaves could occur (Oishi 2013). For four-ring and five-ring PAHs, the significant correlation between PAH accumulation coefficient in plant material vs. the PAH content in sewage sludge was observed. This supports the supposition that uptake of four- and five-ring PAHs occurs via roots. The correlation for four- and five-ring PAHs (Fig. 2 and 3) was observed irrespective of the compound, its origin, and its content in sewage sludge and also irrespective of the absorbing plant type. In the case of three-ring PAHs, the situation is differentiated depending on the plant type (Fig. 1). For Monocotyledones, most probably the root uptake mechanism does occur, confirmed by the existence of a significant correlation between the content of fluorene, phenanthrene, and anthracene in plant material and in sewage sludge. For Dicotyledones, the observed correlation was insignificant. In this case, the mechanism of uptake by leaves could occur or two mechanisms could occur simultaneously—the uptake by roots and by leaves. Both studied plant species belonging to Monocotyledones are characterized by very narrow leaves of small surface area, whereas the leaves of both studied species belonging to the group of Dicotyledones have a larger surface area. It cannot be excluded that three-ring PAHs are absorbed both by plants via roots and from the air via stomata (Meudec et al. 2006; Duarte-Davidson and Jones 1996). As volatile compounds, the three-ring polyaromatic hydrocarbons can be easily absorbed by plants from the air. These hydrocarbons can originally occur in the atmospheric air or appear by evaporation from sewage sludge. The question is why anthracene seemed to be taken up by roots both by Monocotyledones and Dicotyledones, as the only one three-ring PAH. It may be caused by its being different from the physicochemical properties of other three-ring PAHs. Older data indicated lower solubility of anthracene in water (0.23–0.447 μmol/L in 25 °C) in comparison with phenanthrene (5.58–9.0 μmol/L in 25 °C) (Pearlman et al. 1984). New sources show a reversed situation—solubility of anthracene in water (73 μg/L in 25 °C) seems to be higher than solubility of phenanthrene (1.29 × 10^−3^ μg/L in 25 °C) and fluorene (1.98 × 10^−3^ μg/L in 25 °C) (Gupta et al. 2015). Vapour pressure of these compound is also significantly different. It may be assumed that for easier soluble anthracene, the mechanism via root uptake may occur, which is confirmed in the significant correlation between the content of anthracene in sewage sludge and its accumulation coefficient. For phenanthrene and fluorene, which are more volatile and less soluble, the mechanism of uptake of PAHs via leaves from the air may occur, coexisting or not with the mechanism of uptake via root.

**Table 2:** Results of regression analysis between accumulation coefficient of PAH in plant material (Y) vs. the content of PAH in sewage sludge (X)

	Dicotyledones (*n* = 9)^a^				Monocotyledones (*n* = 10)			
	a	b	*R* ^2^	*P*	a	b	*R* ^2^	*P*
Three-ring hydrocarbons								
Fluorene	0.231	−0.319	5.7	0.569	0.157	−0.778	69.4	0.003
Phenanthrene	0.149	−0.240	1.2	0.781	0.285	−0.786	76.5	<0.001
Anthracene	0.019	−0.838	57.0	0.030	0.030	−0.718	73.3	0.002
Four-ring hydrocarbons								
Fluoranthene	0.123	−1.053	92.4	<0.001	0.147	−0.848	95.8	<0.001
Pyrene	0.081	−1.019	78.7	0.001	0.076	−0.932	89.1	<0.001
Benzo[a]anthracene	0.009	−0.829	80.8	0.002	0.008	−1.137	86.7	<0.001
Chrysene	0.017	−0.837	90.9	<0.001	0.018	−1.002	86.2	<0.001
Five-ring hydrocarbons								
Benzo[b]fluoranthene	0.011	−0.934	87.9	<0.001	0.011	−0.905	77.3	<0.001
Benzo[k]fluoranthene	0.006	−0.841	84.7	<0.001	0.006	−0.830	83.7	<0.001
Benzo[a]pyrene	0.009	−0.930	83.6	<0.001	0.008	−1.069	59.9	0.009
Dibenz/ah/anthracene	0.002	−0.933	80.5	0.001	0.004	−0.591	54.2	0.015
Six-ring hydrocarbons								
Benzo[ghi]perylene	0.005	−0.906	71.7	0.004	0.007	−0.959	69.0	0.003
Indeno[123-cd]pyrene	0.005	−0.956	88.8	<0.001	0.004	−0.408	16.5	0.245

Applied regression model: Y = aX^b^

*R* ^*2*^ coefficient of determination (%), *P* observed significance level for *F* test, of which a value below 0.05 indicates significant correlation

^a^In some cases, sample size was equal to 8 because of missing data

It is also unclear, why the significant correlation between the accumulation coefficient of indeno[123-cd]pyrene in the plant material vs. the PAH content in sewage sludge has been observed only for plants belonging to the Dicotyledones group (Fig. 4). This also needs further investigations. For benzo[ghi]perylene, the second analyzed aromatic hydrocarbon with six rings, a significant correlation between the accumulation coefficient in plant material vs. the content of it in sewage sludge was observed for both studied plant groups (Fig. 4).

Nevertheless, the existence of the correlation between the PAH content in plant material and in the substrate they are grown in seems to be unquestionnable. The correlation is of significant character without any doubts. The studies have to be continued for four- and five-ring PAH series. Most of polyaromatics influencing human health belong to this group. The following PAHs proved to have significant correlation, irrespective of the type of plant and the origin of the sewage sludge: benzo[a]pyrene, classified as human carcinogen (group 1); dibenz[ah]anthracene classified as probable human carcinogen (group 2A); and benzo[a]anthracene, benzo[b]fluoranthene, benzo[k]fluoranthene, and chrysene, classified as possible human carcinogens (group 2B).

## Conclusions

The results of the experiment carried out on plants growing spontaneously on sewage sludge, under natural conditions simulating the agricultural uses, demonstrated that the uptake of PAHs by a plant depended on polyaromatic hydrocarbon concentration in sewage sludge. The relation between PAH accumulation coefficient in plant material vs. the PAH content in sewage sludge was of exponential character. The results indicate that for four- and five-ring PAHs, the root uptake mechanism from soil solution occurs, irrespective of the type and origin of sewage sludge and the content of PAHs in it and irrespective of the type of plant. A significant correlation was observed for both, Monocotyledones and Dicotyledones, and it did not depend on the municipal or petrochemical origin of sewage sludge the plants were growing in. For three-ring PAH absorption, the root uptake mechanism occurred in case of Monocotyledones, which is confirmed by the existence of a significant correlation between the content of fluorene, phenanthrene, and anthracene in plant material and in the sewage sludge. For Dicotyledones, the correlation is of insignificant character. In this case, probably two competitive mechanisms occur—the PAH uptake by roots and dissolved three-ring PAHs’ absorption by leaves. The difference between the uptake of PAHs by two groups of plants was demonstrated also for six-ring PAHs. The correlation between the content of PAHs in sewage sludge and in plant material was significant for the Dicotyledones group. Further investigations are needed in order to explain why the correlation between the content of PAHs in sewage sludge and in plant material for Dicotyledones is significant for the group of four-, five-, and six-ring PAHs and in case of Monocotyledones for three-, four-, and five-ring PAHs. For the group of PAHs which is the most important and most dangerous to human health, four- and five-ring PAHs, which are the group including known human carcinogen such as benzo[a]pyrene, probable human carcinogen dibenz[a,h]anthracene, and possible human carcinogens benzo[a]anthracene, benzo[b]fluoranthene, benzo[k]fluoranthene, and chrysene, the correlation between the content of PAHs in sewage sludge and in plant material was significant for both groups of plants, Monocotyledones and Dicotyledones, irrespective of the municipal or industrial origin of sewage sludge.
